# How Accurate Are Population Predictions? Wind Farms and Egyptian Vultures as a Case Study

**DOI:** 10.3390/biology14121743

**Published:** 2025-12-05

**Authors:** Miguel Ferrer, Jorge García-Macía, Mar Sánchez, Virginia Morandini

**Affiliations:** 1 Estación Biológica de Doñana, CSIC, Avda. Américo Vespucio s/n, 41092 Sevilla, Spain; 2 Fundación Migres, CIMA, N-340 km 85, 11380 Tarifa, Spain

**Keywords:** PVA, viability of population, wind farm, bird mortality, *Neophron percnopterus*, prediction of population trajectories, observed versus predicted population trajectories

## Abstract

It is clear that scientists’ predictions must be rigorous and based on scientific evidence, but, even more, it is crucial to review scientific predictions after a reasonable time. However, predictions of published PVAs have rarely been contrasted with real populations’ trends over time. This is worrisome because this is the only way to keep learning and improve our ability to make more accurate predictions. In addition, conservation efforts can shift the initial predictions for the viability of threatened populations; thus, the evaluation of initial predictions becomes required over time. This is the case of the Egyptian vulture in Spain, where trajectories of real populations over the years differ from large-scale predictions. Its extinction in the Iberian Peninsula—due to mortality in wind farms, among other causes—was predicted by 2020, according to published viability analyses; yet, 14 years after this publication, not only did it not happen, but its national (and European) population remains stable and is even slightly increasing (+2.6%). These differences between predicted and observed trajectories of populations show the limitations of the simulations as a conservation tool and offer the opportunity to evaluate the used PVAs and the shortcomings that affected the assessment of the real trajectory of the species. With only four years of data available to simulate and generate 100-year predictions, along with the distribution and variance of mortality rates by collision in wind farms and an overestimation the number of pairs in risk areas, a clear relationship was assumed between predicted risk according to distances and the actual recorded mortality at wind farms, even though it is known that these are not closely related.

## Simple Summary

Population viability analysis is a modeling tool estimating the future size and risk of extinction for populations. Population viability analyses are widely used in setting conservation policies. There is disagreement about the precision of this method. To gain a clearer picture of the usefulness of population viability analysis packages, many more predicted population trajectories need to be evaluated. This will provide vital feedback information that can be used to refine the models, which should in turn produce increasingly accurate predictions of population viability. The objective of this work was to analyze discrepancies between predictions and reality of a population viability analysis for the Egyptian vulture population. Real and additional data have shown that the population viability analysis predictions, i.e., the complete extinction of the Egyptian vulture by those dates, did not come true, fortunately; in fact, the conservation status of the species in question has improved. We discuss reasons for that discrepancy and how to avoid these mistakes in the future.

## 1. Introduction

The need to constantly review our understanding of endangered species’ threats cannot be underestimated. In fact, conservationists should be encouraged to undertake such regular work to ensure that, firstly, when threats have been removed and/or mitigated, such work is appropriately acknowledged. Secondly, to celebrate the successes when a species’ conservation status has improved, and thirdly, when additional data are available, to recognize that perhaps the initial concerns were incorrect.

Population viability analysis (PVA) is a modeling tool that estimates the future size and risk of extinction for the populations of organisms [1,2,3]. PVA is a way to predict the probability of population extinction by inputting actual life-history information and projecting it forward using stochastic computer simulation [4,5]. Although population viability analysis (PVA) is widely used and can be extremely useful in setting conservation policies, there is disagreement about the precision of this method. Objections have been raised concerning the precision of predictions, sometimes in view of the short time series of data available and the effects on the estimated parameters [6,7,8,9]. A review of the use of demographic models for endangered species management [5] pointed out that poor data cause difficulties in parameter estimation, which in turn lead to unreliable estimates of extinction risk. To gain a clearer picture of the usefulness of PVA packages, many more predicted population trajectories need to be evaluated. This feedback process is fundamental to evaluate the real utility of this approach as a useful tool for species conservation, especially in setting conservation policies. We need much more information about the real accuracy of past PVAs by confronting predicted versus observed trajectories in different species and populations. Only this process will provide us with vital feedback information that can be used to refine the models, which should in turn produce increasingly accurate predictions of population viability. This is the objective of this work.

In 2009, a paper dealing with the long-term effect of wind farm mortality on the viability of the Egyptian vulture population (*Neophron percnopterus*) in Spain was published [10]. Wind farms receive public and governmental support as an alternative energy source that mitigates air pollution and facilitates the transition to the decarbonization of society. With 61,460 MW of installed power as of 12 November 2020 and 56.04% of total installed power, Spain is one of the leading countries in the world [11] in the production of energy from renewable sources per capita. Developing renewable energy has become a common strategy and effort in countries all over the world, since it became fundamental to reverse global climate change [12]. Wind power is one of the oldest and most developed sources of renewable energy, with an increase in production exceeding 20% annually over the past decade [13]. In accordance with the current environmental issue, wind facilities generate a low environmental impact by reducing environmental pollution and water consumption [14].

However, it is known that wind farms can have adverse effects on wildlife, particularly through bird and bat collision with rotating turbine rotor blades. Bats and birds are the most impacted taxa because, as flying animals, they are more prone to collide with the turbine rotor [15,16,17,18,19]. Low collision rates have been registered in some studies, such as 0.001 birds/turbine/year [20], but also high collision rates have been reported in others (1.33/turbine/year), with the griffon vulture (*Gyps fulvus*) being the most frequently killed raptor species (0.41 deaths/turbine/year) [17,21]. These findings indicate that mortality rates per turbine are quite variable because the probability of collisions depends on a range of factors such as species, species-specific flight behavior, weather, and topography around wind turbines [15,16,21,22,23]. There is a consensus that raptors may be more prone to colliding with blades than other birds [24] due to their morphology, foraging behavior, or flight behavior [25,26]. Wind farms are assumed to have an important impact on raptor populations, because raptors have longer life spans and lower reproductive rates, resulting in population growth rates that are highly sensitive to adult and subadult mortality [27,28,29].

## 2. Materials and Methods

**Figure 1:**
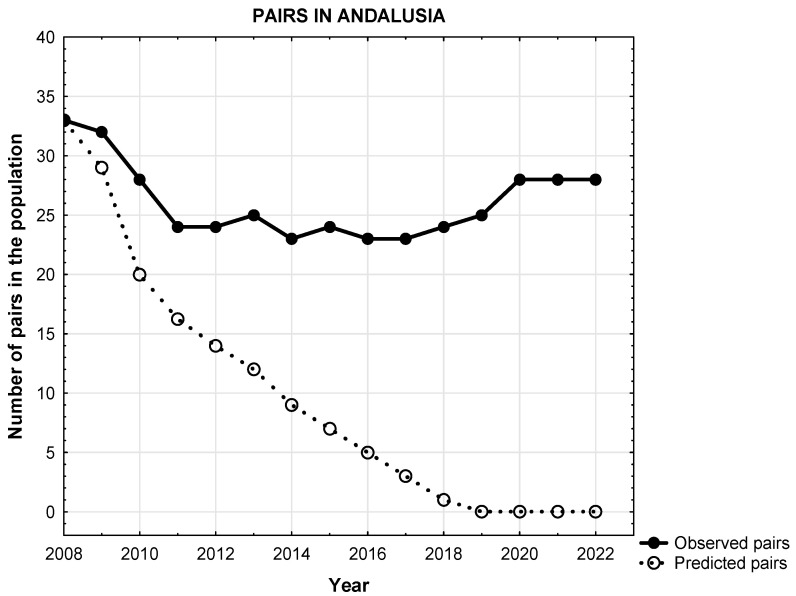
Predicted (according to [10]) versus observed trajectories for the Southern population of Egyptian vultures. As we can see, trajectories differ dramatically. In fact, ref. [10] predicted complete extinction of the species from the Southern population (Andalusia) by 2018. Breeding pairs and years are presented in the axis.

**Figure 2:**
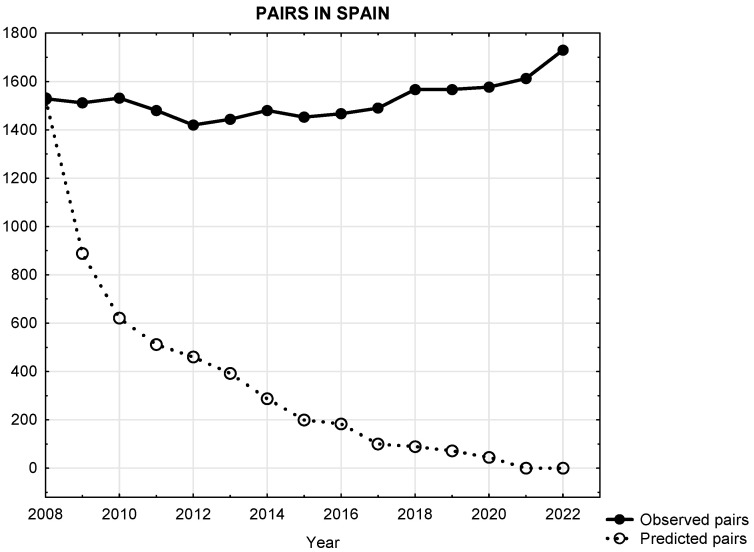
Predicted (according [10]) versus observed trajectories for the Iberian Peninsula population of Egyptian vultures. As we can see, trajectories differ dramatically. In fact, ref. [10] predicted complete extinction of the species from the Iberian Peninsula by 2021. On the contrary, this population experienced an increase of 2.6%. Breeding pairs and years are presented in the axis.

To compare predicted and observed trajectories of populations of Egyptian vultures, we used published information on real historical trajectories according to national censuses [30] and other online information [31]. Using all the available data, we present in Figure 1 and Figure 2, the real trajectories of both Spanish and the Southern Egyptian vulture populations.

In addition, we calculated the real values of some demographic parameters during the 2009–2022 period, now available from the same sources as above, and compared them with the ones available during the 2004–2008 period, which were used to construct the PVA used in [10]. We recorded and analyzed the results reported by the plan against poison, developed by the Andalusia Environmental Ministry, looking for some trend in this source of non-natural mortality, which was the most important for the species at the beginning of this century [32].

The Egyptian vulture is a medium-sized, cliff-nesting, trans-Saharan migrant raptor that defends long-term established territories during the breeding season. Most territories hold a single nest (rarely 2–3 nests situated in the same or adjacent cliffs) that is occupied year after year over long periods of time. The long-term monitoring of marked birds shows that territories are reoccupied every year in early March by their previous owners or, when one dies, by a replacement bird [10]. Most territories hold a single nest (rarely 2–3 nests situated in the same or adjacent cliffs) that is occupied year after year over long periods of time. The dispersal of breeders is rare (only in 7.5% of 203 breeding attempts did one of the birds change territory). In the few areas where species numbers have recovered, most of the territories consist of recolonizations of vacant territories (the so-called extinct territories referred to throughout the text). Therefore, territorial extinctions occur not as a result of breeder dispersal, but because an external cause has affected the territory owners and/or the territorial ‘attractiveness’ for recruitment.

Despite being a territorial breeder, this species may forage and roost socially. This is especially true for non-breeding birds, which also migrate to and from Europe and gather in communal roosts during the breeding season. These roost sites are located near predictable food sources such as rubbish dumps or muladares (places traditionally used by farmers for dumping dead livestock).

In Andalusia, a total of 269 wind turbines from 20 wind farms were monitored from 2008 to 2022 to determine Egyptian vulture collisions. Eleven collided individuals of the Egyptian vulture were found during the non-migratory period, which we assume belonged to local population. These wind farms were constructed and began operation between 2006 and 2007, being the same wind farms studied for estimated Egyptian vulture mortality [10]. According to environmental impact (EID) regulations, facilities were required to develop surveillance programs [16,33]. The main goal of these programs is to document all the mortalities caused by the collisions of birds with turbine blades. These programs have been conducted annually every day of the year (365 days) from dawn to dusk (between 8 and 14 daylight hours in winter and summer, respectively) by 12–17 trained observers who are interconnected by cellular telephones. Mortality searches are carried out at all turbines on a daily basis, with a fixed search effort to find dead birds or bats. The observers are evenly distributed throughout the area covered by the wind farms. With this high search effort, we do not need to apply corrections for search efficiency and scavenger removal, particularly in the case of a large bird like the Egyptian vultures. Anyway, our main goal was to detect relative trends in mortality. These data enable us to determine how mortalities were distributed among the different wind farms and their turbines.

After 2007, the selective stopping of turbines by observers when dangerous situations are detected became mandatory in Andalusia [18]. In these cases, the observers telephone the wind farm control office to switch off the turbines involved in the risk, stopping the turbine within a maximum of 3 min. All 20 wind farms studied have been required to carry out this stopping procedure since 2008 for all medium to large-sized birds that were observed in a risk situation. As a result, we were able to obtain data from the 269 wind turbines from 2008 to 2022 (14 years). Data were recorded according to the protocol designed by the Migres Foundation and collected by the Andalusia Environmental Ministry. We use these data as a proxy to detect a potential change (increase or decrease) in Egyptian vulture mortality rates.

### 2.1. Supplementary Feeding

Supplementary feeding is a common practice to increment the reproductive output in raptors and other bird species, either for experimental or for conservation purposes (e.g., California condor, sparrowhawk, various vulture species, common kestrel, and bearded vulture) [34,35,36,37,38,39,40]. Despite the widespread use of this technique over the past 50 years, particularly in endangered species, its application in conservation has only recently been critically discussed [41], revealing important differences in the evaluation of the technique. Some claim major beneficial effects at the population level, but others report little or no effect [39,42,43].

Significant positive effect of supplementary feeding in the case of the Egyptian vulture has been reported [40,44,45]. Typically, food is placed every day or two, close to the nests of selected pairs for part or all of the breeding cycle from before laying to independence of young, depending on the objective. The usual aims are to increase the size of the clutch or prevent nestling deaths by increasing the nutritional condition of adults.

### 2.2. Simulations

We conducted a simulation using Vortex 10, including the same simulation inputs as other authors [10], but substituting the values they used for both productivity and adult mortality recorded during the 2004–2022 period (Table 1). Following the criteria proposed by other authors [46], simulations were run during twice the life span of the species, 50 years in this case. We conducted simulations to analyze the evolution of predicted and observed population under different scenarios. We used the Vortex simulation software (Vortex, version 10.0.76). Vortex is an individual-based model for population viability analyses (PVAs). It models population dynamics as discrete, sequential events that occur according to probabilities defined by the user, and it can model constant or random variables that follow specified distributions. The events used for modeling describe the typical life cycle of sexually reproducing diploid organisms. This method is particularly appropriate for species showing low fecundity, long life span, small population size, estimable age-specific fecundity and survival rates, and monogamous breeding, as in the species and populations we modeled here. In fact, Vortex has already been used to analyze the viability of populations of Egyptian vultures [10]. We conducted several simulations for different scenarios, performing 1000 replicates for each one.

## 3. Results

### 3.1. Predicted Versus Observed Population Trajectories of Egyptian Vultures Subsection

**Figure 3:**
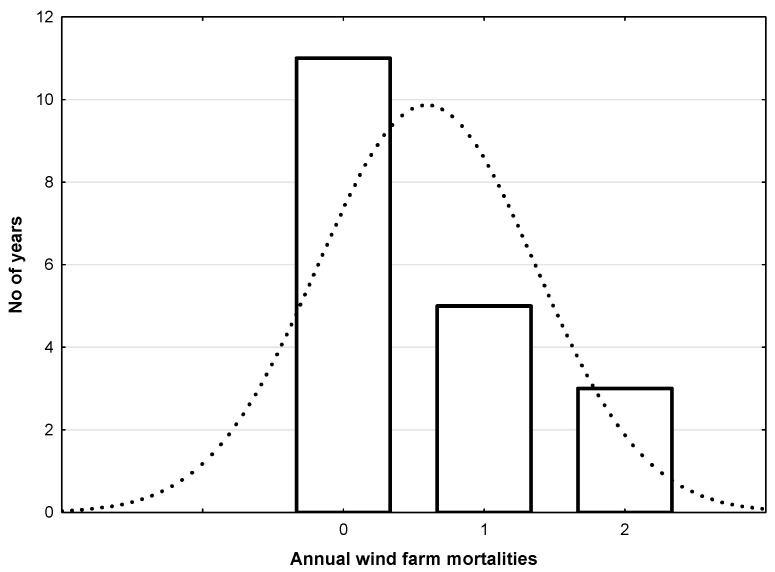
Wind farm annual mortality of Egyptian vultures in Andalusian population during 2004–2022. This mortality follows a Poisson distribution (lambda = 0.57, Kolmogorov–Smirnov d = 0.56; X^2^ = 0.535; *p* = 0.464) and not a normal one (dotted line; Kolmogorov–Smirnov d = 0.35; X^2^ = 105.419; *p* < 0.001).

The Egyptian vulture in Spain, whose extinction in the Iberian Peninsula is due, among other causes, to mortality in wind farms, was predicted by 2020 (according to [10]); and yet, 14 years after this publication, its national (and European) population remains stable and even slightly increasing (+2.6%) [30]. According to [10], considering wind-farm-induced mortality in the species, all simulations performed showed highly negative stochastic growth rates (see Figure 3 [10]), with values lower than 0. In fact, their results predicted complete extinction of the Southern population by 2018 (Figure 1) and, some years later, by 2021, the complete extinction of the species from the Iberian Peninsula (Figure 2).

In the Egyptian vulture metapopulation, i.e., the Iberian population, and following the results of their simulations (see Figure 3 in [10]), the predicted negative slope was −0.8683, decreasing from 1529 breeding pairs in 2008 to 0 pairs in 2021. However, the real observed trajectory of the Iberian population has shown positive slope of +0.7860, increasing from 1529 breeding pairs in 2008 to 1612 pairs in 2021.

A similar pattern can be found in predicted versus observed trajectories for the Andalusian population, which they called the Southern population (see Figure 3 in [10]). The predicted slope was −0.9386, decreasing from 33 breeding pairs in 2008 to 0 pairs in 2021. However, the observed slope has been −0.6747, decreasing from 33 breeding pairs in 2008 to 28 pairs in 2021.

These differences between predicted and observed trajectories of populations offer a unique opportunity to review the initial variables included and the construction of the PVA to evaluate the underlying causes of those differences.

### 3.2. Change in Baseline Conditions

#### 3.2.1. Mortality by Wind Farms

In [10], four years of data were used to estimate mortality rates due to wind farms (2004–2008) for the Andalusian population, and intervals of confidence were not considered for simulations. Within this period of data, the mean mortality of Egyptian vultures due to wind farms obtained was 0.8/year (Table 1). Considering the data now available, from 2009 to 2022, the real mean has been 0.50/year (Table 1). Consequently, mortality from wind turbines recorded during these years has been 1.6 times lower in the Southern population than the one predicted by [10]. Considering the full studied period, from 2004 to 2022, the mean has been 0.57/year (Table 1). Consequently, mortality from wind turbines recorded during these years has been 1.4 times lower in the Southern population than the one predicted by [10]. Those differences between estimated parameters, which seriously affect predictions, partially explain the differences found between predicted and observed trajectories.

#### 3.2.2. Productivity

We have been providing supplementary feeding in Andalusia since 2017. Supplementary feeding is aimed at particular territorial pairs within the Southern population, and it is performed for only a limited period each year. The results showed an important variation in mean fertility over the years. Other authors [10] recorded a mean productivity of 0.633 during the period 2004–2008 (Table 1). Considering the data now available from 2009 to 2022, the mean has been 0.947 (Table 1), that is, 1.49 times greater than the one used by [10] in their predictions. For the full studied period (2004–2022), mean productivity was 0.864, which is 1.36 times greater.

#### 3.2.3. Mortality from Poisons

Unfortunately, the use of poisoned baits is still a widespread practice in Spain and Europe to control natural predators of game and livestock species. This nonselective method poses a deadly threat to many threatened species in Andalusia such as the Spanish imperial eagle, the bearded vulture, or the Egyptian vulture. Despite the complexity of the actions required for its eradication, the measures implemented under the Andalusian Strategy against Poison in 2004 have led to a 50% reduction in the use of poisons in the region, from 205 cases detected in 2006 to 94 in 2012, and only 21 cases in 2019 (89.75% reduction). These measures placed Andalusia at the forefront of the fight against poison in Europe.

Adult poison mortality was 0.019 during the 2004–2008 period in Andalusia, decreasing to 0.009 during the period 2009–2022. For all the study period (2004–2022), the mean mortality for this cause was 0.002, which is 9.5 times lower than the one used in the simulation by [10].

### 3.3. Distribution of Simulated Demographic Stochasticity and Environmental Variability

Demographic stochasticity is the random fluctuation in the observed birth rate, death rate, and sex ratio of a population resulting from stochastic sampling processes. Environmental variability, on the other hand, is the annual fluctuation in probabilities of birth and death arising from random fluctuations in the environment. Demographic stochasticity is commonly modeled in VORTEX and other programs as binomial or, optionally, beta distributions, while environmental variation is modeled frequently as a normal distribution. Mortality rates, especially due to human infrastructures, tend to usually show non-normal distributions, following a strong Poisson distribution instead. Remarkably, the spatial pattern of mortality found in wind farms [16,18,21], resembling that caused by other anthropogenic infrastructures such as power lines [47] or roads [48], shows that few pylons, road sections, or wind turbines typically account for the majority of casualties. Therefore, the identification and subsequent application of mitigation measures in mortality hotspots would dramatically reduce overall mortality [47,49].

In fact, using homogeneity tests, the annual mortality of Egyptian vultures from the wind farm in the Andalusian population during 2004–2022 follows a Poisson distribution (lambda = 0.57, Kolmogorov–Smirnov d = 0.56; X2 = 0.535; *p* = 0.464) and not a normal one (Kolmogorov–Smirnov d = 0.35; X2 = 105.419; *p* < 0.001, see Figure 3). These particular distributions of mortality at man-made infrastructures tend to be spatially highly concentrated. An additional factor affecting the predictions of [10] has been that data used showed a higher variance than expected in a normal distribution (mean = 0.8; variance 1.2), suffering over dispersion.

### 3.4. Estimation of the Number of Pairs in Risk Areas

From 2004 to 2008, the authors in [10] found two territorial and three non-territorial (two young and one individually marked 6-years-old floater) birds dead at wind farms located in the Andalusian population. The closest distance between the breeding territories suffering from these casualties and the wind farms was 6.37 and 14.57 km, so they decided to use 15 km as a guide radius to obtain wind farm risk zones, assuming that all breeding pairs located within an area of 15 km radius around the wind farms, anywhere in Spain, must have the same risk of collisions. That included 389 pairs in Spain and 7 in the Andalusian population.

According to the paragraph above, these particular distributions of mortality at man-made infrastructures like windfarms tend to be spatially highly concentrated. It has been demonstrated that mortality does not depend on the wind farm but on the exact location of individual wind turbines [16,18,21,22]. According to [22], soaring birds do not move at random over the area, but follow some trajectories more than others. These preferred trajectories were determined by the wind speed, which was in turn related to the underlying topography of individual wind turbines. Consequently, certain locations of wind turbines could show a high collision risk even if there is a relatively low density of birds crossing the area, whereas other locations could be safer even with higher densities of birds in the wider area. This means that there is no clear relationship between predicted risk according to bird densities and actual recorded bird mortality at wind farms [16]. Risk assessment studies incorrectly assumed a linear relationship between the frequency of observed birds and fatalities. However, it is known that bird abundance and bird mortality through collision with wind turbines are not so closely related; this result challenges a frequent assumption, such as that formulated by [10], linking wind farm mortality risk to the distances of Egyptian vulture nests. In this case, predictions would be overestimating mortality rates for more than 400 pairs.

### 3.5. Simulation Results

**Figure 4:**
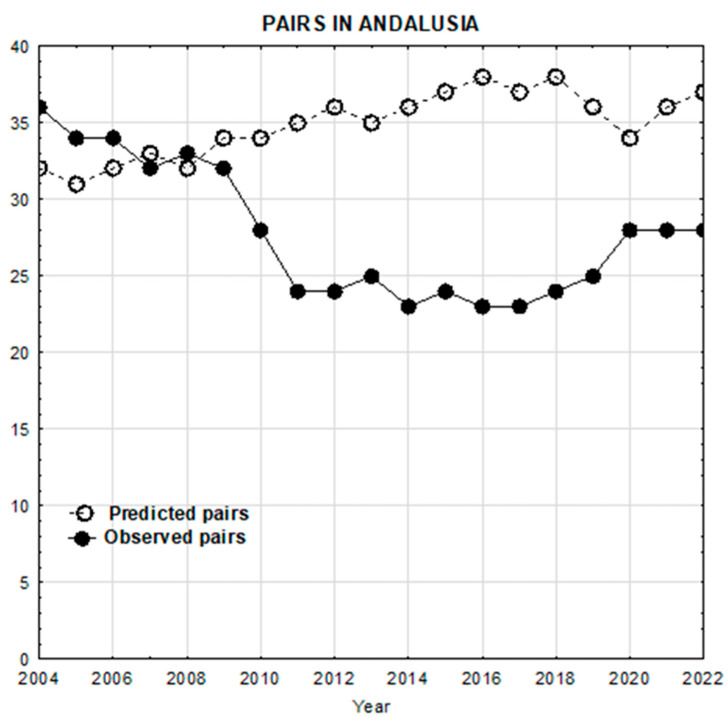
Predicted (according to new simulations) versus observed trajectories for Andalusian population of Egyptian vultures. As we can see, using the new values for productivity and mortality, both trajectories do not differ dramatically.

The probability of extinction of the 100 simulated populations using the new baseline parameters was PE = 0.00. The simulated population showed a stable evolution and even a slightly increasing trend, with a positive stochastic population growth rate (rs = 0.023) and a positive deterministic population growth rate (rd = 0.024), with a λ > 1 (lambda = 1.024), again showing the trend towards a slight increase over years (Figure 4).

## 4. Discussion

Here, we reviewed previously published PVAs [10] warning of the imminent extinction of Egyptian vulture populations in Spain, and particularly in Andalusia. Real and additional data have shown that the PVA predictions had not come true, and, in fact, the conservation status of the species in question has improved.

According to our results, variation in baseline conditions was one of the reasons for these discrepancies between predicted and observed trajectories. Excellent results of the Andalusian Strategy against Poison, which achieved an 89.75% reduction in mean mortality for this cause, which is 9.5 times lower than the one used in the previous simulation [10], is one of the factors explaining differences between predicted and observed trajectories. Increase in fecundity, which was in fact 1.36 times greater than predicted, is in part due to supplementary feeding programs. Mortality from wind turbines recorded during the 2004–2022 period has been 1.4 times lower than the one predicted by [10] for the Southern population. Those differences between estimated parameters seriously affect predictions, partially explaining the differences found between predicted and observed trajectories.

In PVAs, the results depend sensitively on estimated parameters. Short time series or poor fits of the model to data lead to wide confidence intervals for the predicted trajectories of the simulated population. In many cases, the size of the confidence interval is so wide that estimates may become meaningless [9]. For the assessment of PVAs, it is essential to account for imprecision in parameter estimates and its consequences for risk assessment. A variety of tools are available, such as confidence intervals on the risk of extinction within a given time horizon.

Other authors [10] alerted to the potential negative effects of wind farms on the conservation of many endangered species. They predicted future extinction scenarios that never happened. According to our current knowledge, the reasons beneath these differences between predicted observed population trajectories are (i) the amount of initial data, with only four years of data to simulate and generate 100-year predictions, (ii) changes in baseline conditions, with 1.4 times more productivity and lower mortality than estimated, (iii) not considering the actual distribution and its variance in mortality rates by collision in wind farms, and (iv) overestimation of the number of pairs in risk areas, assuming a clear relationship between predicted risk according distances and the actual recorded mortality at wind farms, when the abundance and mortality through collision with wind turbines are not linearly related. Abundance is a covariate, not an explanatory variable, in mortality at human-made infrastructures.

According to [10], current strategies implemented by power companies, based on vigilance of risky areas such that turbines are stopped when birds approach them, are completely inefficient in cases of solitary territorial birds that are rarely detected by observers [10]. However, this strategy, now called a turbine shutdown system, has achieved a 61.7% reduction in mortality of soaring birds and 92.8% specifically of collisions of griffon vultures, showing a net-beneficial application elsewhere [18]. In fact, it seems that this technique, perhaps in conjunction with supplementary feeding programs, was good enough to decrease the mortality of wind turbines by 1.4 times in the case of the Egyptian vulture.

No single wind turbine or wind farm has been removed in Spain to prevent Egyptian vulture collisions from 2008 to 2022, as far as we know, providing no support to an alternative explanation difference between predictions and observed trajectories—i.e., a decrease in the number of risk areas, which would in turn decrease mortality. In fact, in 2009, 19,148.8 MW were installed and operating in Spain, increasing to 29,813 MW by 2022, an increase of 10,664 MW [50]. What really changed during the years, after the predictions in [10], were productivity and adult mortality. The effort of previous authors [10] was important to send a warming message about the status of Egyptian vulture populations, encouraging mitigation actions including both poison and collision with wind turbines, but is also a good example of the limitations of the simulations as a conservation tool. With only four years of data, a long-term simulation is always dangerous due to small sample size to determine the means and variance of key demographic parameters. In this case, the imprecision in those demographic parameters was so high that the simulation results become meaningless.

It is clear that scientists’ predictions must be rigorous and based on scientific evidence, but, even more, it is crucial to review scientific predictions after a reasonable time. However, the quality of published PVA predictions is rarely assessed. This is worrisome, because this is the only way to keep learning and improve our ability to make more accurate predictions.

## 5. Conclusions

This manuscript addresses, in our opinion, an important and often overlooked aspect of conservation science: the need to reassess past population viability predictions in light of subsequent reality. The Egyptian vulture in Spain could be a strong and timely case study. By confronting the forecasts made in 2009 [10] with actual monitoring data, we clearly show that the predicted extinction has not occurred and that, in some cases, population trends have been stable or even slightly positive. This is a valuable message, both for the specific conservation context of the species and for the broader methodological debate on predictive models. The study’s strengths lie in the long-term dataset used, the contrast between modeled and observed trajectories, and the identification of key differences in baseline conditions—such as reduced poisoning, lower wind farm mortality, and higher productivity—that help explain the discrepancy between the 2009 forecasts and reality.

This paper also addresses the question of scientific accountability. This case illustrates not only the limitations of PVA when based on short or incomplete datasets, but also the consequences of publishing forecasts that prove to be seriously inaccurate, especially when they influence conservation policy, funding, and public perception. We may wish to reflect explicitly on the responsibility of scientists to revisit their predictions, acknowledge when they are wrong, and ensure that conservation decisions are based on the best available evidence.

One clear conclusion that we can learn from this study is that PVA predictions must be confronted with real population trajectory when available. The final objective of any PVA is to determine if demography indicates a serious and imminent risk of extinction, but it is not very clear what criteria would be used to define what constitutes such a risk. A major problem with defining a risk of extinction is that, by any criteria, this definition is limited in time. Even taking this into account, we still need some objective criteria to decide when a population is at risk in order to obtain the public support for a long-term conservation action.

The main point for us is not to secure the population indefinitely, but to decide when the population is not at risk. Using simulations and objective criteria, like probability of extinction below 1% and positive trend over twice the lifespan of the species, we can make predictions about the length of the predictions. Predicted trajectories of the simulations can be used to check annually if the evolution of the new population is over, on, or under expectations. Adjusting the simulated period to twice the life span of the species allows us to compare species with different life histories in comparable units of time.

The original assessment [10] has limitations stemming from initial data scarcity, shifts in baseline conditions, failure to account for the actual distribution and variability of mortality risk from turbine collisions, and an overestimation of the number of breeding pairs in risk areas. Evaluating the predictive accuracy of PVAs by contrasting forecasts with observed trends is an essential exercise for model validation, adaptive management, early detection of unforeseen threats or positive actions, and the refinement of future PVAs. These differences between predicted and observed trajectories of populations show the limitations of simulations as a conservation tool and offer the opportunity to evaluate the used PVAs and the shortcomings that affected the assessment of the real trajectory of the species, such amount of initial data—with only four years of data to simulate and generate 100-year predictions—the distribution and variance of mortality rates by collision in wind farms, and the overestimation of the number of pairs in risk areas. Additionally, a clear relationship was assumed between predicted risk based on distances and the actual recorded mortality at wind farms, even though these factors are not closely related.

## Figures and Tables

**Table 1:** Differences in baseline conditions according to period used for estimates. For all the studied period (2004–2022), mean productivity was 0.864, which is 1.36 times greater than estimates used by [10]. Mortality from wind turbines had been 1.4 times lower than the one predicted by [10]. For all the study period (2004–2022), the mean mortality for poison was 0.002, which is 9.5 times lower than the one used in the simulation by [10]. Period 2004–2008 was the time period used to estimate demographic parameter by other authors [10].

Time Period	Fecundity (SD)	Mortality by Wind Turbines (SD)	Poison-Related Mortality (SD)
Period 2004–2008	0.633 (0.074)	0.80 (1.095)	0.019 (0.02)
Period 2009–2022	0.947 (0.460)	0.50 (0.650)	0.009 (0.09)
Total 2004–2022	0.864 (0.418)	0.57 (0.768)	0.002 (0.02)

## Data Availability

The raw data supporting the conclusions of this article will be made available upon request.
